# Mitophagy switches cell death from apoptosis to necrosis in NSCLC cells treated with oncolytic measles virus

**DOI:** 10.18632/oncotarget.2028

**Published:** 2014-05-28

**Authors:** Mao Xia, Gang Meng, Aiqin Jiang, Aiping Chen, Meike Dahlhaus, Patrick Gonzalez, Christian Beltinger, Jiwu Wei

**Affiliations:** ^1^ Jiangsu Key Laboratory of Molecular Medicine, Medical School and the State Key Laboratory of Pharmaceutical Biotechnology, Nanjing University, Nanjing, China; ^2^ Nanjing University Hightech Institute at Suzhou, Suzhou, China; ^3^ Dept. of Pediatrics and Adolescent Medicine, University Medical Center Ulm, Ulm, Germany; ^4^ Inserm U785, Centre hepato-biliaire, Hopital Paul Brousse, Villejuif, France

**Keywords:** oncolytic measles virus, non-small cell lung cancer, autophagy, mitophagy, apoptosis, necrosis

## Abstract

Although apoptotic phenomena have been observed in malignant cells infected by measles virus vaccine strain Edmonston B (MV-Edm), the precise oncolytic mechanisms are poorly defined. In this study we found that MV-Edm induced autophagy and sequestosome 1-mediated mitophagy leading to decreased cytochrome c release, which blocked the pro-apoptotic cascade in non-small cell lung cancer cells (NSCLCs). The decrease of apoptosis by mitophagy favored viral replication. Persistent viral replication sustained by autophagy ultimately resulted in necrotic cell death due to ATP depletion. Importantly, when autophagy was impaired in NSCLCs MV-Edm-induced cell death was significantly abrogated despite of increased apoptosis. Taken together, our results define a novel oncolytic mechanism by which mitophagy switches cell death from apoptosis to more efficient necrosis in NSCLCs following MV-Edm infection. This provides a foundation for future improvement of oncolytic virotherapy or antiviral therapy.

## INTRODUCTION

The attenuated measles virus of the Edmonston strain lineage B (MV-Edm) is a promising oncolytic single-stranded RNA virus that has entered clinical trials [1, 2]. However, the precise mechanisms underlying MV-Edm-induced cell death have not yet been well defined and require further clarification. This is not only crucial for oncolytic virotherapy, but also of great interest for understanding the infectious cytopathy of measles virus.

Cell death can be classified into three major types: apoptosis, autophgic cell death, and necrosis in accordance with its morphological appearance [3]. Apoptosis is a process of programmed cell death characterized by unique morphological changes including nuclear fragmentation, chromatin condensation, plasma membrane blebbing, and cytoskeletal disassembly [4, 5]. Autophagy is a fundamentally homeostatic process that allows cells to recycle their components and remove damaged organelles. A hallmark in this process is the formation of vesicles called autophagosomes cargoing components for lysosomal degradation. The selective autophagy targeting impaired mitochondria is known as mitophagy [6]. While autophagy is critical for cell survival under stress conditions, persistent or immoderate autophagy can result in autophagic cell death (also referred to type-II cell death), which is characterized by the accumulation of autophagosomes (autophagic vacuoles) in the cytosol without appearance of apoptosis [7-9].

Autophagosomal formation can be triggered by MV-Edm at a very early stage of infection through a CD46-Cyt-1/GOPC pathway [10]. Furthermore, MV-Edm sustains autophagy for viral infectivity [11]. Measles virus has also been found to subvert the autophagy network for its replication by an IRGM-dependent pathway [12, 13]. Our more recent work shows that MV-Edm utilizes mitophagy to promote viral replication by mitigating antiviral innate immune responses [14]. Interestingly, as some apoptotic characteristics have been observed in MV-Edm infected cancer cells reported by several previous studies [15-19], recent work shows that MV-Edm-induced autophagy protects malignant cells from apoptosis [11]. However, how virus-induced autophagy counteracts apoptotic pathways remains unclear.

Various crosstalks between autophagy and apoptosis have been identified [20-23]. Little is known about the contribution of autophagy and its crosstalk with apoptosis in solid tumors treated with MV-Edm. In this study, we investigated the role of autophagy in regulation of MV-Edm-induced cell death and its crosstalk with apoptosis in NSCLCs. We show that mitophagy switches cell death from apoptosis to more efficient necrosis in NSCLCs following MV-Edm infection.

## RESULTS

### MV-Edm induces autophagy and preserves autophagic flux

**Figure 1:**
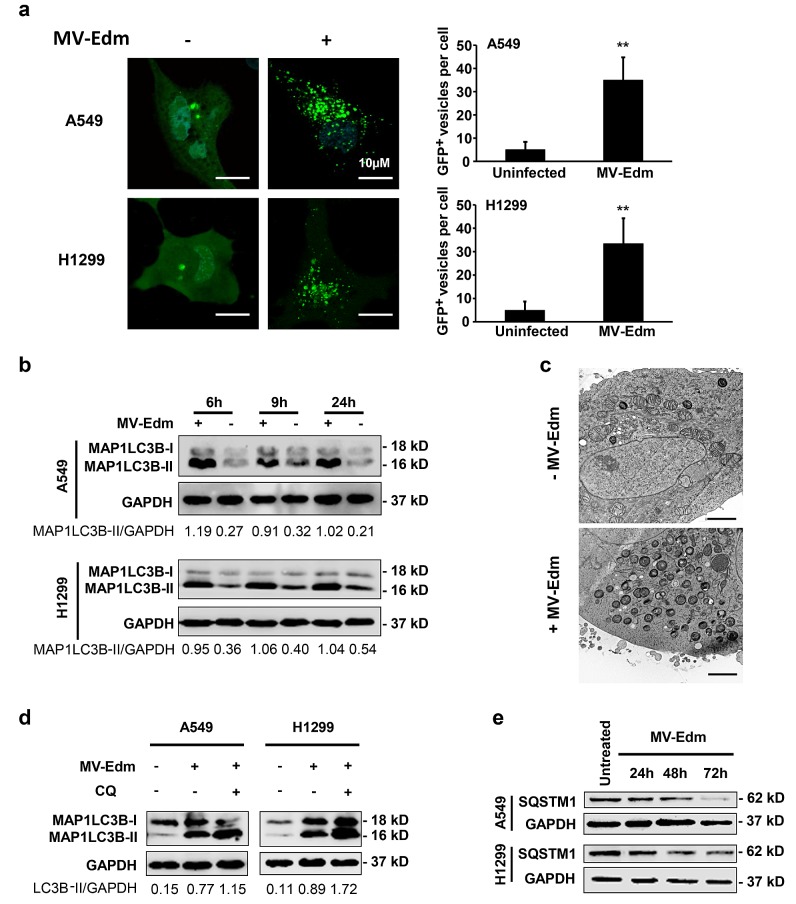
MV-Edm infection induces autophagy and preserves autophagic flux (a) A549 and H1299 cells were transiently transfected with a plasmid encoding EGFP-LC3 for 24 h and further incubated for 6 h with or without MV-Edm (MOI = 0.5). Aggregation of EGFP-MAP1LC3B at autophagosomes was evaluated by fluorescence confocal microscopy. EGFP^+^ vesicles (green dots) of each cell represent autophagosomes (left panel) were quantified (right panel). Bars represent 10 μm. (b) Levels of lipidated MAP1LC3B were assessed by Western blot in lysates obtained from A549 and H1299 lung cancer cells infected with MV-Edm at a MOI of 0.5 or left uninfected for 6, 9 and 24 hours. Densitometry is shown as the ratio of MAP1LC3B-II to GAPDH. Similar results were obtained in three independent experiments. (c) A549 cells were infected with MV-Edm at a MOI of 1 for 24 h (lower panel), or left uninfected (upper panel). Cells were then subjected to electron microscopy for detection of autophagosomes. Double- or multilayered-structures with intracellular contents are shown in MV-Edm-infected. Bars represent 2 μm. (d) A549 and H1299 cells were infected with MV-Edm at a MOI of 0.5 for 4 h and grown in the absence or presence of chloroquine (CQ, 40 μM) for another 5 h before cell lysates were harvested for Western blot. Densitometry of MAP1LC3B lipidation is shown as the ratio of MAP1LC3B-II to GAPDH. Blots are representative of two independent experiments. (e) A549 and H1299 cells were infected with MV-Edm at a MOI of 0.5 for 24, 48 and 72 h. Cell lysates were then harvested for Western blot against SQSTM1. Representative blots from two independent experiments are shown. ** p < 0.01.

Several recent studies have shown that MV-Edm induces autophagy both at early and late stage following infection [11, 24]. First we wanted to confirm the autophagic response to MV-Edm infection in NSCLCs. By overexpressing the enhanced green fluorescent protein (EGFP) - microtubule-associated protein 1 light chain 3 beta (MAP1LC3B/LC3) in NSCLCs, we found that puncta of EGFP-MAP1LC3B increased in the cytosol in infected cells compared to the uninfected group (Fig. 1a). We confirmed autophagy by detecting the conversion of MAP1LC3B-I to MAP1LC3B-II by Western blot. Cells with MV-Edm infection showed an increased amount of MAP1LC3B-II in cell lysates when compared with uninfected cells (Fig. 1b). In line, we observed that massive double- or multi-layered structures containing intracellular contents occurred in A549 cells infected with MV-Edm (Fig. 1c). To determine whether MV-Edm infection enhances autophagic flux, we analyzed both lipidation of MAP1LC3B in presence of chloroquine and expression of SQSTM1. SQSTM1 is a marker of late stage autophagy that is degraded together with the contents of autophagosomes upon their fusion with lysosomes. As shown in Fig. 1d, levels of lipidated MAP1LC3B were further increased when infected cells were treated with chloroquine, a well-known acidification inhibitor that inhibits the activity of the pH-dependent lysosomal protease resulting in blockage of autolysosomal degradation [25]. Moreover, SQSTM1 expression started to decrease 48 h after MV-Edm infection, which was consistent with degradation of SQSTM1 via enhanced preserved autophagic flux (Fig. 1e). These results indicate that MV-Edm induces the complete autophagic process by means of enhancing the autophagic flux in NSCLCs.

### Autophagy is correlated with MV-Edm-mediated oncolysis in NSCLCs

**Figure 2:**
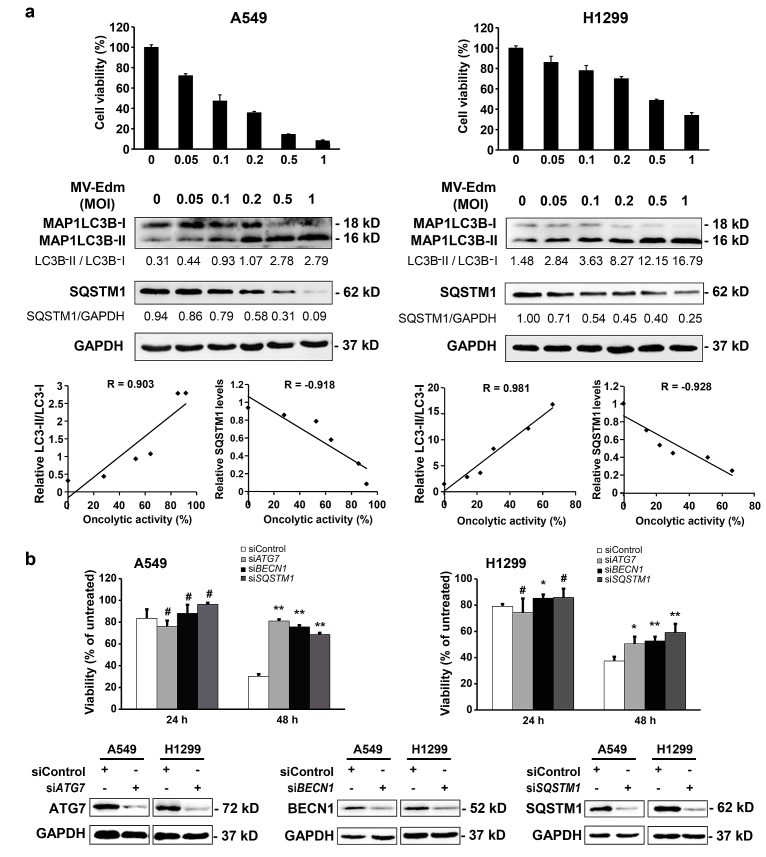
Autophagy contributes to MV-Edm-induced oncolysis (a) A549 and H1299 cells were infected with MV-Edm at MOIs as indicated for 72 h. Cell death (upper panels) was measured by trypan-blue exclusion, MAP1LC3B-II conversion and SQSTM1 levels (middle panels) were determined by Western blot. Correlation between cell death and autophagy-related markers was analyzed by the Excel 2010 Analysis ToolPak (lower panels). R > 0.8 means strong correlation. Similar results were obtained in two independent experiments. (b) A549 and H1299 cells were transfected with siRNA targeting ATG7, BECN1, SQSTM1, or with non-targeting control siRNA for 24 h, and then infected with MV-Edm at a MOI of 0.5 for another 24 and 48 h, respectively. Cell death was quantified by trypan-blue exclusion (upper panel). Similar results were obtained in three independent experiments. Quality control of gene silencing was monitored by Western blot (lower panel). A representative result from two independent experiments is shown.

The oncolytic mechanisms of MV-Edm are not well clarified. We next wanted to know the role of autophagy in MV-Edm-mediated cell death. To evaluate the relationship between MV-Edm-induced autophagy and oncolytic effect as described [26], cell viability and autophagic changes, i.e. conversion of MAP1LC3B-I to MAP1LC3B-II and downregulation of SQSTM1 proteins, were analyzed [27]. Significant correlations between MV-Edm-induced cell death and the autophagy-related markers, such as LC3B-II/LC3B-I ratio and SQSTM1/p62 level were observed in A549 and H1299 cells (Fig. 2a), suggesting a relationship between induction of autophagy and oncolytic activity following MV-Edm infection. To further confirm the role of autophagy in MV-Edm-induced cell death, we blocked the proteins essential for autophagy by RNAi against ATG7 or BECN1. As shown in Fig. 2b, cell death was significantly decreased in autophagy-impaired cells following MV-Edm infection. Taken together, these results clearly show that autophagy contributes to MV-Edm-mediated oncolysis in NSCLCs.

### Autophagy blocks caspase-dependent apoptosis leading to enhanced viral replication in MV-Edm-infected NSCLCs

**Figure 3:**
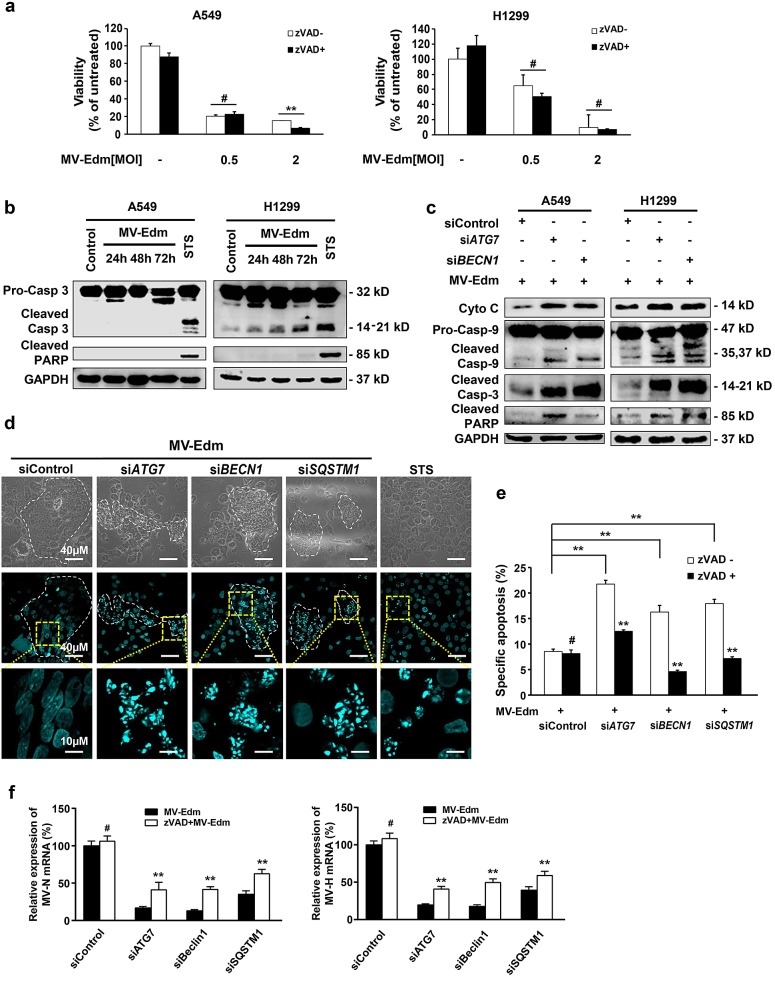
Autophagy protects NSCLCs from apoptosis leading to enhanced viral replication (a) A549 and H1299 cells were infected with MV-Edm at a MOI of 0.5 or 2 in the absence or presence of a pan-caspase inhibitor z-VAD-fmk (80μM) for 72 h. Cell viability was quantified by trypan-blue exclusion. Means + SD of triplicates are shown. Similar results were obtained in three independent experiments. (b) A549 and H1299 cells were infected with MV-Edm at a MOI of 0.5 for 24, 48 and 72 h. Cell lysates were harvested for Western blot. Cell lysates from untreated and staurosporin (STS) treated cells were used as negative and positive controls, respectively. Representative blots from two independent experiments are shown. (c) Cytoplasmic cytochrome, cleaved caspase -9, -3 and PARP were evaluated by Western blotting cell lysates harvested from A549 and H1299 cells transfected with siRNAs targeting ATG7, BECN1 or non-specific control siRNA followed by MV-Edm infection at a MOI of 0.5 for 48 h. A representative result from two independent experiments is shown. (d) A549 cells were transfected with siRNA targeting ATG7, BECN1, SQSTM1, or with non-targeting control siRNA for 24 h followed by infection with MV-Edm (MOI = 0.5) for another 48 h. Cells were stained by DAPI before subjected to fluorescent confocal microscopy for evaluation of fragmented nuclei (blue spots). Cells treated with staurosporin (STS, 500 nM) for 12 h were used as a positive control. Scale bars represent 40 μm (upper two panels) and 10 μm (lower panel). Dashed white lines highlight multinucleated syncytia. (e) A549 cells were transfected with siRNA targeting ATG7, BECN1, SQSTM1, or with non-targeting control siRNA for 24 h followed by infection with MV-Edm (MOI = 0.5) in the presence or absence of zVAD (80 μM) for another 48 h. Cells were harvested and the specific apoptosis was analyzed by determining hypodiploid nuclei by FACS. Means + SD of triplicates are shown. Similar results were obtained in three independent experiments. (f) A549 cells were transfected with siRNAs targeting ATG7, BECN1, SQSTM1 or non-specific control siRNA for 24 h followed by infection with MV-Edm (MOI = 0.5) in the absence or presence of zVAD (80 μM) for another 48 h. Then the expression of N- and H-viral structural genes was quantified by qRT-PCR. Means + SD of triplicates are shown. Similar results were obtained in three independent experiments. # p > 0.05, * p < 0.05, ** p < 0.01.

Some apoptotic characteristics were found in MV-Edm-infected glioma and breast cancer cells in several previous studies [15-17]. However, none have investigated the contribution of apoptosis compared to other modes of cell death in MV-Edm-mediated oncolysis. To our surprise, while MV-Edm induced cell death in NSCLCs, this effect could not be blocked by the pan-caspase inhibitor z-Vad-fmk (Fig. 3a), indicating that predominant cell death in MV-Edm-infected cells might not be caspase-dependent apoptosis. In line with this, cleaved PARP and caspase 3 were not increased in A549 cells, and only mildly increased in H1299 cells even 72 h after MV-Edm infection (Fig. 3b). However, the cleaved caspase-3, caspase-9 and PARP were markedly increased in autophagy knockdown cells following MV-Edm infection (Fig. 3c). Moreover, we found that autophagy was involved in modulating cytochrome c release from mitochondrion, as cytochrome c release into the cytoplasm was strongly increased in autophagy knockdown cells (Fig. 3c). Interestingly, we found that nuclear fragmentations typical for apoptosis following MV-Edm infection occurred exclussively in autophagy knockdown cells treated with ATG7, BECN1 and SQSTM1 siRNA, respectively, whereas nuclei within syncytia remained intact in control siRNA treated cells (Fig. 3d). Of note, the nuclear fragments were all confined to syncytia, the typical cytopathy induced by MV-Edm infection. In line, specific apoptosis quantified by flow cytometry was significantly increased in autophagy-impaired cells, and could be abrogated by z-VAD-fmk (Fig. 3e). These data clearly show that autophagy blocks caspase-dependent apoptosis. To further clarify the relationship among autophagy, apoptosis and viral replication, we determined viral replication in autophagy knockdown cells in the presence or absence of z-VAD-fmk. While N- and H-protein gene expressions were massively decreased in autophagy-impaired cells, they were significantly albeit partially rescued in the presence of z-VAD-fmk (Fig. 3f). Together, these data suggest that autophagy inhibits caspase-dependent apoptosis and thus favors viral replication.

### Mitophagy counteracts apoptotic cascade by controlling cytochrome c release

**Figure 4:**
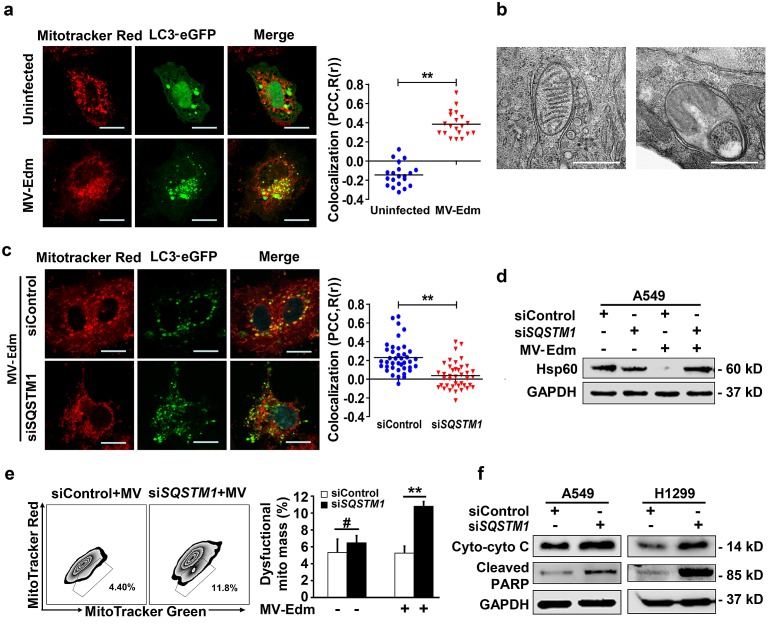
MV-Edm induces mitophagy to control cytochrome C release in NSCLCs (a) A549 cell were transiently transfected with a plasmid encoding EGFP-MAP1LC3B for 24 h and infected with MV-Edm at a MOI of 1 for another 12 h, or left uninfected. Cells were then stained with Mitotracker red and subjected to confocal microscopy. Scale bars represent 10 μm. Colocalization (yellow dots) of mitochondria (red) with autophagosomes (green puncta) was quantified by calculating Pearson's correlation coefficient (PCC, R(r)) (right panel). Means are shown (n = 30 of each). (b) Subcellular structures were analyzed by electron microscopy in cells infected with MV-Edm at a MOI of 1 for 24 h. Left panel shows a double-layered membrane engulfing a mitochondrion (early stage of mitophagy), and right panel shows a double-layered membrane containing a degraded mitochondrion (late stage of mitophagy). Bars represent 0.5 μm. ** indicates p < 0.01. (c) Colocalization of autophagosomes and mitochondria was quantified in A549 cells transfected with SQSTM1 siRNA for 24 h followed by transfection with pEGFP-Map1lc3b for another 24 h. Cells were then infected with MV-Edm at a MOI of 0.5 for 12 h and stained with Mitotracker Red before subjected to confocal microscopy (left panel). Scale bars represent 10 μm. Colocalization (yellow dots) of mitochondria (red) with autophagosomes (green puncta) was quantified by calculating Pearson's correlation coefficient (PCC, R(r)) (right panel). Means are shown (n = 30 of each). (D & E) A549 cells transfected with siRNA targeting SQSTM1, or a non-specific control siRNA for 24 h. Then cells were infected with MV-Edm at a MOI of 0.5 for another 48 h, or left uninfected. (d) The cell lysates were harvested and the mitochondrial HSPD1 (Hsp60) protein levels were determined by Western blot. A representative result from two independent experiments is shown. (e) Cells were incubated with mitotracker green and red before subjected to FACS analysis. Gated subpopulations depict damaged mitochondria (left panel). Percent of injured mitochondria was quantified (right panel). Means + SD of 3 independent experiments are shown. (f) A549 and H1299 cells were transfected with siRNA targeting SQSTM1 or non-specific control siRNA for 24 h, cells were then infected with MV-Edm at a MOI of 0.5 for another 48 h. Cytoplasmatic cytochrome c and cleaved PARP were evaluated by Western blot. A representative result from two independent experiments is shown. # p > 0.05, * p < 0.05, ** p < 0.01.

As shown above, cytochrome c release was markedly increased in autophagy-impaired cells following MV-Edm infection. Next we investigated the mechanisms by which MV-Edm-induced autophagy interferes with the apoptotic pathway. As we previously reported that MV-Edm induced mitophagy in NSCLCs, we reasoned that mitophagy might contribute to restrict cytochrome c release. First, we confirmed that MV-Edm triggered mitophagy in NSCLCs, as colocalization of lipidated MAP1LC3B with mitochondria was massively increased following MV-Edm infection (Fig. 4a). This was further confirmed by electron microscopy. Shown is a mitochondrion was trapped in a double-layered membrane (Fig. 4b, left panel), and a mitochondrion degraded in an autophagosome (Fig. 4b, right panel). And we also confirmed that MV-Edm-induced mitophagy was mediated by SQSTM1, as colocalization of lipidated MAP1LC3B with mitochondria was impaired by silencing SQSTM1, a key mediator cargos mitochondrion to autophagosome (Fig. 4c). Moreover, we found that HSP60 (a conservative mitochondrial protein) was markedly decreased following MV-Edm infection, however, it was massively preserved in SQSTM1 knockdown cells (Fig. 4d). Next we found that the dysfunctional mitochondria significantly accumulated in SQSTM1 knockdown cells infected with MV-Edm, but not in uninfected cell (Fig. 4e), suggesting that mitophagy plays a crucial role in clearance of damaged mitochondria. Lastly, we found that impaired mitophagy by SQSTM1 knockdown led to cytochrome c release and PARP cleavage (Fig. 4f). Taken together, these data suggest that MV-Edm utilizes mitophagy to counteract apoptosis by preventing cytochrome c release into the cytoplasm.

### Oncolytic MV-Edm induces necrosis in NSCLCs

**Figure 5:**
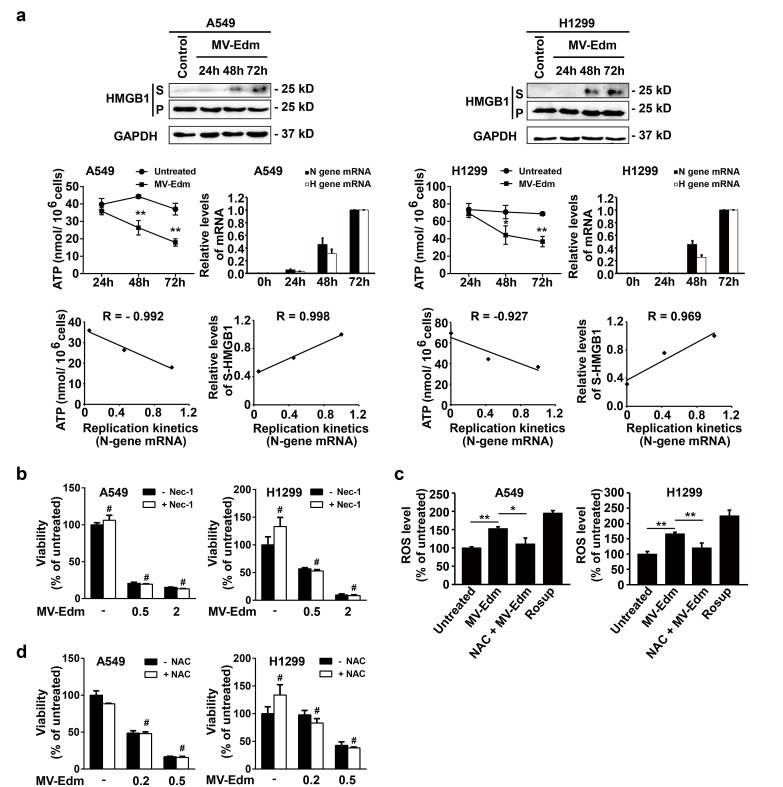
MV-Edm infection induces necrotic features in NSCLCs (a) A549 and H1299 cells were infected with MV-Edm at a MOI of 0.5 for 24, 48 and 72 h. Supernatants (S) and cell pellets (P) were then harvested for determination of necrosis-related marker HMGB1 by Western blot (upper panel). Intracellular ATP levels were quantified by a luciferase based ATP kit and viral replication was quantified by qRT-PCR of viral structural N- and H-protein genes (middle panel). Correlations of MV-Edm replication (viral structural N protein genes) with ATP content, and with the HMGB1 were analyzed by the Excel 2010 Analysis ToolPak (lower panel). Similar results were obtained in two independent experiments. (b) A549 and H1299 cells were pretreated by the Rip-1 inhibitor necrostatin-1 (50 μM) for 1 h followed by infection with MV-Edm at a MOI of 0, 0.5 and 2 for another 48 h. Cell viability was quantified by trypan blue exclusion. Similar results were obtained in two independent experiments. (c) A549 and H1299 cells were infected with MV-Edm at a MOI of 0.5 in the presence or absence of 5 mM NAC for 6 h. Cells were then stained by DCFH-DA and subjected to flow cytometry for evaluation of ROS generation. Cells treated with Rosup for 20 minutes and untreated cells were used as positive and negative controls, respectively. (d) A549 and H1299 cells were infected with MV-Edm at a MOI of 0, 0.2 and 0.5 in the presence or absence 5 mM NAC for 48 h. Cell viability was determined by trypan blue exclusion. Means + SD of triplicates are shown. Similar results were obtained in three independent experiments. # p > 0.05, * p < 0.05, ** p < 0.01.

Having shown that autophagy inhibited apoptosis and in parallel enhanced oncolysis, we next sought to determine which type of cell death might predominantly contribute to MV-Edm-mediated oncolysis. As shown in Fig. 5a, viral replication increased after infection with MV-Edm for 48 and 72 h, and concomitantly cellular ATP levels started to decrease after 48 and 72 h in NSCLCs. There was a significant correlation between ATP reduction and viral replication (Fig. 5a). Furthermore, we found that the high mobility group box 1 protein (HMGB1), a marker of necrosis as cell membranes rapture [28], was markedly increased in the supernatant of MV-Edm-infected cells 48 and 72 h post-infection. Again, there was a significant correlation between viral replication and HMGB1 release (Fig. 5a). These results suggest that ongoing viral replication resulted in ATP exhausting consequently leading to necrosis. The pharmacological inhibitor of necroptosis (necrostatin-1, an inhibitor of RIPK1), failed to prevent cell death by MV-Edm in NSCLCs (Fig. 5b), excluding the possibility of programmed necrosis mediated by the RIP-1 [29]. Furthermore, we found that ROS generation was increased following MV-Edm infection and could be blocked by an antioxidant NAC in NSCLCs (Fig. 5c). However, NAC failed to block MV-Edm-induced cell death, which excluded the possibility of ROS-mediated necrosis (Fig. 5d). Taken together, these data suggest that MV-Edm induces necrosis due to persistent ATP consumption and exhaustion along with viral replication.

## DISCUSSION

The precise mechanisms of oncolysis by MV-Edm remain unclear. Here we show that MV-Edm utilizes mitophagy to counteract apoptosis by controlling cytochrome c release. Decreased apoptosis in line favors persistent viral replication leading to dominant necrotic cell death due to ATP exhaustion. Our study sheds light on how MV-Edm induces oncolysis. It provides a rationale for future improvement of MV-Edm-based oncolytic virotherapy.

### Death of NSCLCs induced by MV-Edm is not apoptotic

Apoptosis has been considered to be the predominant mechanism induced by oncolytic MV-Edm in solid tumors [15, 17]. However, the relative contribution of apoptosis to MV-Edm-induced oncolysis remained unclear. To our surprise, we found that cell death was not inhibited by the pan-caspase inhibitor zVAD-fmk in NSCLC cells infected by MV-Edm. Lack of apoptosis was further supported by the absence of activation of caspase-3 and subsequent absence of PARP cleavage. Given that MV-Edm killed 50 – 70% of cells infected, these observations suggest that its oncolytic effect on NSCLCs may not rely on apoptosis, despite the limited presence of apoptotic features. In fact, the apoptotic markers observed in aforementioned studies occurred at a relatively late stage after MV-Edm infection. For instance, while more than 70% of glioma cells were dead 2 d after MV-Edm infection, syncytia remained TUNEL-negative [17]. In another study, the increase of TUNEL-positive cells, activated caspases and cleaved apoptotic substrates, were observed on day 4 after MV-Edm infection [15], while massive cell death had occurred before. Thus, other mechanisms beyond apoptosis might exist in MV-Edm-mediated oncolysis.

### Mitophagy controls cytochrome c release and inhibits apoptosis leading to enhanced viral replication

The crosstalk between autophagy and apoptosis has been intensively studied in recent years. Accumulated evidences show that autophagy is cytoprotective in cells under stress and reduces the tendency of cells undergo apoptosis [20, 30-32]. In line, our results showed that caspase-dependent apoptosis occurred exclusively in autophagy-impaired NSCLCs following MV-Edm infection. A recent study also showed that MV-Edm-induced autophagy in HeLa prevented apoptosis [11], however, it remains unclear how autophagy counteracts apoptosis and how autophagy contributes to MV-Edm-induced oncolysis. We found that MV-Edm induced SQSTM1-mediated mitophagy, crucial for clearance of damaged mitochondria before cytochrome c is released. Thus, our results show that MV-Edm uses mitophagy to control cytochrome c release and to subsequently prevent downstream caspase activation. However, our results do not rule out that MV-Edm utilizes autophagy to counteract apoptosis by other means. This requires further intensive investigations.

Of note, the autophagic adaptor SQSTM1, which is recruited to damaged mitochondria and is essential for their final clearance by autophagic degradation [33], was markedly decreased 2 d and almost disappeared 3 d after MV-Edm infection. This suggests that sustained autophagy exhausts intracellular protein required for autophagic flux. We postulate that SQSTM1 exhaustion might promote pro-apoptotic signaling and indeed, knockdown of SQSTM1 expression resulted in significant increase of apoptosis even at an early stage (2 d) after MV-Edm infection. These results may explain the observations in previous studies that apoptotic features occurred at a relatively late stage following MV-Edm infection [15-17].

Apoptosis is regarded as one of the fundamental mechanisms by which hosts defend themselves against viral infection [34-36]. Since self-destruction may restrict viral replication and spread, it is plausible that MV-Edm utilizes mitophagy to counteract apoptosis and thus to favor its replication. This assumption was confirmed by the fact that in autophagy-impaired NSCLCs, inhibition of apoptosis by z-VAD-fmk enhanced viral propagation. Noteworthy, mitochondrial degradation by mitophagy also decreased the level of mitochondrion-associated antiviral sensor (MAVS), which abrogated antiviral innate immune responses leading to enhanced viral replication [14].

### Sustained autophagy allows persistent viral replication leading to necrosis due to ATP exhaustion

Surprisingly, despite counteracting apoptotic cell death autophagy correlate with MV-Edm-induced cell death. Oncolysis was abrogated in autophagy-impaired NSCLCs, suggesting that autophagy participated in the regulation of MV-Edm-triggered cell death. Moreover, we found that viral replication of MV-Edm was correlated with ATP exhaustion and with HMGB1 release, suggesting that persistent MV-Edm results in necrosis. We have excluded the possibilities of both RIP1-mediated programmed necrosis and ROS-mediated necrosis. This and given that autophagy promotes MV-Edm infectivity, as shown in this and previous studies [11, 12], we can conclude that autophagy contributes to oncolysis by sustaining viral propagation leading to necrosis because of ATP exhaustion. It remains to be shown whether this mechanism is active in cancer cells other than NSCLCs.

These findings are important for improving oncolytic virotherapy using MV-Edm. Moreover, they might also be beneficial for designing rational antiviral therapeutic strategies, such as using autophagy inhibitors.

## MATERIALS AND METHODS

### Cells, plasmids and siRNA

Human lung adenocarcinoma cell lines A549 (CCL-185), H1299 (CRL-5803) and Vero African green monkey kidney cells (CCL-81) were obtained from American Type Culture Collection (ATCC, Manassas, VA). Cells were maintained in DMEM supplemented with 0.1mM non-essential amino acids, 5% fetal bovine serum, and penicillin-streptomycin (all from Invitrogen, Carlsbad, CA). Expression plasmid: pBABEpuro-EGFP-LC3 (Addgene, #22405) was provided by Jayanta Debnath (University of California, San Francisco). Chloroquine (CQ, C6628) and Necrostatin-1(Nec-1, N9037) were purchased from Sigma-Aldrich (St. Louis, MO). Staurosporin (STS, 11055682001) was purchased from Roche. Z-VAD-fmk (161401-82-7) was purchased from Merck. SiRNA directed against SQSTM1/p62 (NM_003900) was synthesized by GenePharma (Sense, GGAGCACGGAGGGAAAAGAtt; Antisense, UCUUUUCCCUCCGUGCUCCac). The siRNA targeting ATG7 (Invitrogen, HSS116182), BECN1 (Invitrogen, HSS112731), and negative control siRNA (Invitrogen, 12935400) were all purchased from Invitrogen Stealth RNAi collection.

### Viruses

MV-Edm and MV-Edm expressing a reporter gene luciferase (MV-Edm-luc, kindly provided by S. Russell, Mayo Clinic, MN, USA) were propagated in Vero cells with a MOI of 0.02 in 2 ml OptiMEM (Invitrogen, 31985-062) at 37°C for 3 h. The medium was changed to DMEM supplemented with 2% FCS and cells were incubated at 37°C for 1 day before being transferred to 32°C for another day. Cells were harvested and viral particles were released by two cycles of snap freezing in liquid nitrogen and thawing in 37°C water bath. Viral titers were determined by 50% end-point dilution assays (TCID_50_) on Vero cells.

### Transfection

100 nM of siRNA or 500 ng/ml expression plasmids coupled with Lipofectamine 2000 (Invitrogen, 11668-019) were used for transfection of A549 or H1299 cells on a 6- or 12-well plate according to the manufacturer's instructions.

### Cell viability assay

Cells were harvested by trypsin/EDTA and stained with trypan blue, viability was determined by trypan blue exclusion assay.

### Western blot analysis

Cells were lysed in RIPA buffer containing a protease inhibitor cocktail (Roche, 11873580001, South San Francisco, CA). Protein concentration was determined using the bicinchoninic acid assay (BCA assay). Equal amounts of protein were separated by SDS-PAGE and electrophoretically transferred onto a PVDF membrane (Roche, 03010040001). After blocking with 5% nonfat milk in Tris-buffered saline containing 0.1% Tween-20 the membrane was incubated with specific primary antibodies, followed by incubation with appropriate horseradish peroxidase–conjugated secondary antibodies. Signals were detected using an enhanced chemiluminescence reagent (Millipore, WBKLS0500, UK) and subjected to Alpha Innotech Flour Chem-FC2 imaging system (Palo Alto, CA). Antibodies used in this experiment were: anti-GAPDH (Bioworld, MB001, 1:5000 diluted, Nanjing, China), anti-MAP1LC3B/LC3 (Thermo Scientific, PAI-16930, 1:500 diluted, San Jose, CA,), anti-SQSTM1/p62 (Abcam, ab109012, 1:5000 diluted, Cambridge, UK), anti-PARP/Cleaved p85 (Epitomics, #1074-1, diluted 1:400, Burlingame, CA), anti-Cytochrome c (Epitomics, #2119-1, diluted 1:1000), anti-Caspase-3 (Imgenex, IMG-144A, diluted 1:500), anti-Caspase-9 (Cell Signaling Technology, #9502, diluted 1:500, Danvers, MA), anti-ATG7 (Epitomics, #2054-1, diluted 1:10000), anti-BECN1 (Epitomics, #2026-1, diluted 1:500), anti-HSPD1/HSP60 (Epitomics, #1724-1, diluted 1:10000), and anti-HMGB-1 (Abcam, ab18256, diluted 1:500).

For assessment of HMGB-1 release, 2 ml cell supernatant was concentrated by centrifugation via Amicon 3K Ultra-4 centrifugal filter units (Millipore, UFC800308) at 4,300 rpm for 20 minutes.

### ATP assay

Adenosine triphosphate (ATP) was measured by ATP Assay Kit (Beyotime, S0026). Briefly, protein extracts were suspended in standard reaction buffer containing luciferin and luciferase according to the manufacturer's instructions, and luminescence read at 560 nm.

### Quantitative RT-PCR

For quantitative RT-PCR (qPCR), total cellular RNA was extracted with TRIZOL (Invitrogen, 15596-026) and 1μg of RNA was reverse-transcribed using the synthesis system (TaKaRa, DRR036A, Otsu, Shiga, Japan). qPCR was performed using the Real-Time PCR system (ABI 7300, Foster City, CA). Gene expression was calculated with the comparative Ct method and normalized to the endogenous levels of GAPDH. Primer sequences used for qPCR were: 5'-ACATTAGCATCTGAACTCGGTATCAC and 3'-TTTTCGCTTTGATCACC for a 146-bp fragment of MV-Edm N-protein gene; 5'-GATGACAAGTTGCGAATGGAGA and 3'- GACAAGACCCCGTATGAAGGAA for a 125-bp fragment of MV-Edm H-protein gene; 5'- CCATGTTCGTCATGGGTGTGAACCA and 3'- GCCAGTAGAGGCAGGGATGATGTTC for a 251-bp fragment of the human glyceraldehyde-3-phosphatase dehydrogenase gene (GAPDH).

### Confocal microscopy and immunofluorescence

The pBABEpuro-EGFP-LC3/Map1lc3b [37] construct was transiently expressed in A549 or H1299 cells 24 h prior to MV-Edm infection. For some experiments, cells were stained with MitoTracker Red (Invitrogen, M7512) at a concentration of 100nM for 20 min at 37°C in culture medium and then fixed with 4% paraformaldehyde. Cells were observed under a confocal microscope (Olympus, Tokyo, Japan) and images were obtained by digital camera using FV10-ASW software (Olympus) and analyzed with ImageJ (National Institutes of Health, Maryland). Pearson's Correlation Coefficient was quantified using ImageJ software in 30 different cells for each condition.

### Flow cytometric analyses

Pretreated cells were stained by MitoTracker Green (Invitrogen, M7514) and MitoTracker Red (Invitrogen, M7512) according to the manufacturer's instruction. Cells were then washed with PBS solution, trypsinized, and resuspended in PBS solution for FACS analysis (Becton Dickinson, Oxford, UK). Mitochondrial mass was measured by fluorescence intensity of cells.

### Apoptosis assay

Apoptosis was assessed by quantifying DNA fragmentation using FACS analysis (FACScan and FlowJo software, Becton Dickinson) counting hypodiploid (sub-G1) propidium iodide (Sigma, P4170)-stained nuclei as described [38].

### Cell fractionation

Mitochondrial and cytoplasmic proteins were separated using a commercially available Mitochondria/Cytosol Fractionation Kit (Beyotime, C3601, Beijing, China) according to the manufacturer's protocol. Briefly, cells were harvested and washed twice with ice-cold PBS. They were then incubated in 500μL ice-cold mitochondrial lysis buffer on ice for 10 min. Cell suspension was then taken into a glass homogenizer and homogenized for 32 strokes using a tight pestle on ice. The homogenate was centrifuged at 800 × g for 10 min at 4°C to remove any unbroken cells. And the supernatant was further centrifuged at 8000 × g for 10 min at 4°C to obtain the mitochondrial fraction (pellet) and cytoplasmic proteins (supernatant). Samples of mitochondria were dissolved in lysis buffer and proteins were subjected to immunoblotting.

### Electron microscopy

5 × 10^4^cells/cm^2^ A549 cells were seeded on saphire discs (Brügger, Minusio, Switzerland) in a 12-well plate and infected with with MV-Edm at a MOI of 1 for 3 h. Cells were washed and incubated for additional 9 h. Samples were frozen under high pressure, dehydrated and chemically fixed. Ultra-thin section were cut and stained with uranyl acetate and lead citrate. Cells were imaged using a Zeiss EM 10 transmission electron microscope (Berlin, Germany) using an acceleration voltage of 80 kV.

### Statistical analyses

Student t test was used for all statistical analyses.
